# Research Competencies of Registered Pediatric Nurses: Evidence from a Greek Pediatric Hospital

**DOI:** 10.3390/nursrep16010024

**Published:** 2026-01-12

**Authors:** Maria I. Giantsiou, Aristoula Tzalidi, Efrosini Vlachioti, Anastasia A. Mallidou

**Affiliations:** 1 Children’s Hospital Agia Sophia, 11527 Athens, Greece; e.vlaxioti@paidon-agiasofia.gr; 2 Department of Nutrition and Dietetics, University of Thessaly, 42132 Trikala, Greece; aristea.tzalidi@gmail.com; 3 School of Nursing, University of Victoria, Victoria, BC V8N 4V3, Canada; mallidou@uvic.ca

**Keywords:** research competencies, registered nurses, knowledge, skills, attitudes

## Abstract

**Background/Objectives**: The aim of this study was to evaluate the research competencies of pediatric nurses and to assess the psychometric properties of the Research Competencies Assessment Instrument for Nurses (RCAIN) in Greece. **Methods**: A cross-sectional study was conducted in December 2023 via a convenience population-based sample of 106 registered pediatric nurses. Eligible participants owned a diploma, bachelor’s, or graduate degree in nursing and had completed at least two years of professional service. Research competencies were estimated through the RCAIN, a standardized instrument previously validated in the Greek language. **Results**: The findings revealed moderate levels of research-related knowledge (mean score: 26.92/40), skills (mean score: 22.17/30), and application of research in clinical practice (mean score: 14.89/25). Higher educational attainment and participation in scientific activities were positively associated with research competency scores. The RCAIN showed high internal consistency across subscales (Cronbach’s α: knowledge = 0.914, skills = 0.905, application = 0.935), supporting its reliability in this population. **Conclusions**: Pediatric nurses showed moderate research competencies, underscoring the need for direct educational and institutional strategies to foster research capacity and evidence-based practice in pediatric nursing settings.

## 1. Introduction

As a fundamental value of evidence-based nursing practice, research not only guides clinical decision making but also fosters the ongoing scientific growth of the nursing profession [1]. Generally, “research capacity” refers to the ability to combine institutional resources, attitudes, knowledge and skills in order to efficiently conduct, apply and maintain research activities. In nursing, “research capacity” describes nurses’ ability to recognize principles of research, evaluate scientific evidence, lead or participate in research projects and incorporate research findings into clinical practice, thereby advancing the nursing profession and the whole healthcare system, as well as improving patient care [2,3].

However, the numerous and competing demands within healthcare systems have limited the effective implementation of policies designed to strengthen research capacity in nursing [4]. Indeed, even if research is critical in nursing, several studies have revealed that nurses often show insufficient research knowledge, and they usually have little participation in research projects [5,6,7,8]. Insufficient training, lack of institutional support, competing clinical needs and other factors seem to hinder nurses’ engagement in evidence-based practice [9]. In response to these challenges, several countries have included research and evidence-based practice competencies in national nursing education standards, professional competency frameworks, and healthcare policies, recognizing research engagement as a key component of modern nursing roles [10,11,12].

To date, several related instruments have been created to evaluate nursing research competencies, both for undergraduate or doctoral students and for clinical nurses. These instruments are mainly based on nursing research knowledge and skills, attitudes and interest, actions and behaviors that arose from past experiences, values and aspects that cultivate the best nursing practice, the ability to use knowledge for innovative problem solving, applications of research in healthcare, and relevant nurses’ attitudes, as well as nurses’ competencies for evidence-based clinical practice [9]. The Research Competencies Assessment Instrument for Nurses (RCAIN) is a standardized and validated instrument designed to assess nurses’ research-related knowledge, skills, and attitudes, as well as their ability to apply these competencies in clinical practice [9]. RCAIN is grounded in evidence-based practice and competency-based nursing frameworks, conceptualizing research capacity as a multidimensional construct encompassing cognitive, affective, and behavioral fields. By focusing on transferable research competencies applicable across clinical settings, it is particularly appropriate for estimating research competencies among pediatric nurses, whose practice requires the rigorous incorporation of scientific evidence for the care of vulnerable populations [9]. Such instruments are widely used at a global level, not only for individual competency assessments, but also to inform nursing education planning, workforce development, and policy assessment [13,14].

In Greece, pediatric nurses typically acquire research-related knowledge and skills through undergraduate and postgraduate education, as well as voluntary participation in ongoing educational activities, such as scientific meetings and workshops [15]. Formal, structured research training and institutional support in pediatric clinical settings are still restricted, making educational attainment and personal engagement particularly crucial for the evolution of research competencies [15]. History reveals that nursing education in Greece has emphasized clinical skills over research education, with relatively few structured modules on evidence-based practice or research methodologies for undergraduates [15]. Additionally, graduate programs exist, yet they are limited in number and accessibility; moreover, relevant institutional support is poor, often being confined to academic hospitals. As a result, opportunities for nurses in Greece are limited; however, there does not exist a fully established research culture [16]. Therefore, we highlight the need for empirical evaluations of nurses’ research competencies to inform education, professional development, and policy initiatives tailored to the Greek healthcare system. In comparison with international developments, Greek policies aimed at strengthening nurses’ research capacity remain limited, although notable academic initiatives—such as the development of educational programs and validated assessment tools—have emerged to enhance nurses’ research competencies [17]. Nevertheless, despite the existence of such instruments, empirical data on the research competencies of Greek nurses seem absent, especially in specialized healthcare settings such as pediatric hospitals. Therefore, a systematic evaluation is required to identify and better understand strengths and barriers in nursing-research-related domains. Moreover, evidence related to pediatric nurses’ research competences seems scarce. This lack of national empirical evidence hampers the alignment of nursing education, professional development, and healthcare policy in Greece with internationally recognized standards for research and evidence-based practice competencies.

At a conceptual level, the present study is informed by models of nursing research capacity and evidence-based practice development, both of which illustrate research competence as a progressive process evolving from basic research knowledge, to methodological and analytical skills, and, finally, to the application of evidence in clinical practice [2,3]. Such models highlight the role of formal education, ongoing professional development, and organizational support in shaping nurses’ engagement with research [2,3]. In this context, the RCAIN applies research competence across the interrelated domains of knowledge, skills, and application, providing a structured framework for estimating the ways in which research competencies evolve and turn into pediatric nursing practice [9]. The aim of this study is to assess the research capacity of registered pediatric nurses by evaluating their key research competencies, including attitude, skills and knowledge. This study examines the psychometric properties of the RCAIN in a sample of Greek nurses from the largest pediatric hospital in Greece, and it explores variations in research competencies across demographic groups and the associations among the RCAIN subscales.

## 2. Materials and Methods

### 2.1. Survey and the Population-Based Sample

This cross-sectional study was conducted the 2nd half of December 2023 at the largest pediatric hospital in Athens, Greece: Children’s Hospital Agia Sofia. The study was based on the RCAIN, a questionnaire developed by a team of researchers at the University of Victoria, Canada [18].

A convenience sampling method was used, based on staff availability and willingness to participate. Specifically, questionnaires were distributed only to registered nurses who were present on duty during the data collection period; thus, participation was affected by nurses’ workloads, shift schedules, and availability in working hours. The hospital employed 381 registered nurses. In total, 150 paper-based questionnaires were distributed across nursing departments by hand. Five to eight copies were allocated to each department, corresponding to the average number of employed registered nurses. Completed questionnaires were collected one week later, resulting in 106 valid responses, corresponding to an approximate response rate of 27.8%. This sampling approach may have introduced selection bias, since nurses with greater interest in research or professional development could have been more likely to participate. Participants were approached in person and informed about the study procedures and objectives. Participation was solely voluntary, and nurses were assured that declining to participate would not affect their employment or professional assessment. To ensure anonymity, questionnaires were completed with no identifying information and were assigned unique codes. All data were securely stored on password-protected computers accessible only to the research team. Data were analyzed collectively, and findings were presented in summary form in order to protect participants’ confidentiality. The study included registered nurses who held either a diploma or a bachelor’s degree and/or graduate degree in nursing, had completed at least two years of probationary service, and were employed in various nursing roles, including clinical care, leadership, administration, or education. Nurses of all ages, genders, and income levels were eligible to participate. Exclusion criteria were nursing assistants and nursing students currently engaged in clinical training.

### 2.2. The RCAIN

The basic questionnaire of this study included the RCAIN questionnaire and some other basic questions regarding demographic characteristics such as gender, age, marital status, education level, years of work experience, and current unit of employment (e.g., medical, surgical department, pediatric intensive care unit, neonatal intensive care unit, clinical laboratory sector, nursing administration).

The RCAIN questionnaire consists of three sections comprising a total of 19 items. The first section, “knowledge”, includes eight items rated on a 5-point Likert-type scale ranging from (1) “I don’t know at all” to (5) “I know very well (expert)”. The score of this section ranges from eight to 40 (8–40) points. The second section, “skills”, contains six items evaluated on a 5-point scale, from (1) “I am not competent at all” to (5) “I am highly competent (expert)”. The score of this section ranges from six to 30 (6–30) points. The third section, “application of knowledge and skills”, comprises five items, also rated on a 6-point scale, ranging from (1) “I am not knowledgeable and skillful at all” to (5) “I am highly knowledgeable and skillful (expert)”. The score of this section ranges from five to 25 (5–25) points. The total score of the RCAIN ranges from 19 to 95 (19–95) points.

The psychometric properties of the RCAIN have previously been reported, showing strong internal consistency (Cronbach’s α = 0.813–0.946 for each section and 0.937 for the entire tool) and good validity indices, with correlation coefficients ranging from 0.472 to 0.833, RMSEA values below 0.08 (e.g., 0.046), and CFI values above 0.90 (up to 0.96).

### 2.3. Statistical Analysis

Statistical analyses were conducted via IBM SPSS Statistics, version 28.0, for Windows. Data analyses include descriptive statistics, assessment of the RCAIN psychometric properties (i.e., reliability and validity), comparisons of nurse research competencies among groups based on their demographics (i.e., independent *t*-test or non-parametric Mann–Whitney U test, and ANOVA), and correlations among the RCAIN subscales. Descriptive statistics summarize demographic and clinical characteristics. Categorical variables are presented as absolute (n) and relative frequencies (%). Continuous variables are reported as means with Standard Deviations (SD), medians with Interquartile Ranges (IQR), or means with 95% Confidence Intervals (CI), depending on the variable distribution. The Kolmogorov–Smirnov test and histograms were used to assess normality. Cronbach’s alpha coefficients were calculated to assess internal consistency reliability for each of the three RCAIN sections. To assess the construct validity of the questionnaire, a Confirmatory Factor Analysis (CFA) was conducted, during which the following indices were calculated: χ^2^/df, Root Mean Square Error of Approximation (RMSEA), Goodness of Fit Index (GFI), Adjusted Goodness of Fit Index (AGFI), Tucker–Lewis Index (TLI), Incremental Fit Index (IFI), Normed Fit Index (NFI), and Comparative Fit Index (CFI). According to the literature, the acceptable thresholds for these indices should be as follows: χ^2^/df < 5, RMSEA < 0.10, and GFI, AGFI, TLI, IFI, NFI, CFI > 0.90. Thresholds indicating a very good model fit should be: χ^2^/df < 3, RMSEA < 0.08, and GFI, AGFI, TLI, IFI, NFI, CFI > 0.95. Group comparisons among subgroups were performed using either the independent samples t-test or the non-parametric Mann–Whitney U test, depending on data distribution. For comparisons involving more than two groups, Analysis of Variance (ANOVA) or the non-parametric Kruskal–Wallis test was applied. Correlations between continuous variables were assessed using Pearson’s or Spearman’s rho, based on data normality. Homogeneity of variances was evaluated using Levene’s test, and Bonferroni correction was applied to adjust for multiple comparisons among subgroups. Statistical significance was set at *p* = 0.05.

## 3. Results

### 3.1. Demographic Characteristics of the Population-Based Sample

The population-based sample included 106 nurses working at a pediatric hospital. Table 1 presents the demographic characteristics of the participants. Among the 106 nurses, 92 (86.8%) were women with a mean age of 44.20 years (±10.13). Most participants (60.0%) had a diploma in nursing (i.e., graduated from a Technological Educational Institute or TEI). Fourteen participants (13.3%) were master’s students, 30 (28.6%) held a master’s degree, 32 (30.2%) had a nursing specialty, 2 (1.9%) were PhD candidates, and 3 (2.8%) held a doctoral degree. Table 1 illustrates the demographic characteristics of the population-based sample.

### 3.2. RCAIN’s Psychometric Characteristics and Results

The mean score of “knowledge” among participants was 26.92 (±7.08) out of 40 points, reflecting a moderate level of research-related knowledge. The average score of “skills” was 22.17 (±6.33) out of 30 points, indicating moderate to high research skills. The “application of knowledge and skills” had an average score of 14.89 (±5.44) out of 25 points.

The Cronbach’s alpha coefficient for RCAIN’s internal consistency was 0.914 for the “knowledge” section, 0.905 for the “skills” section, and 0.935 for the “application of knowledge and skills” section, indicating the high reliability of the questionnaire.

**Figure 1:**
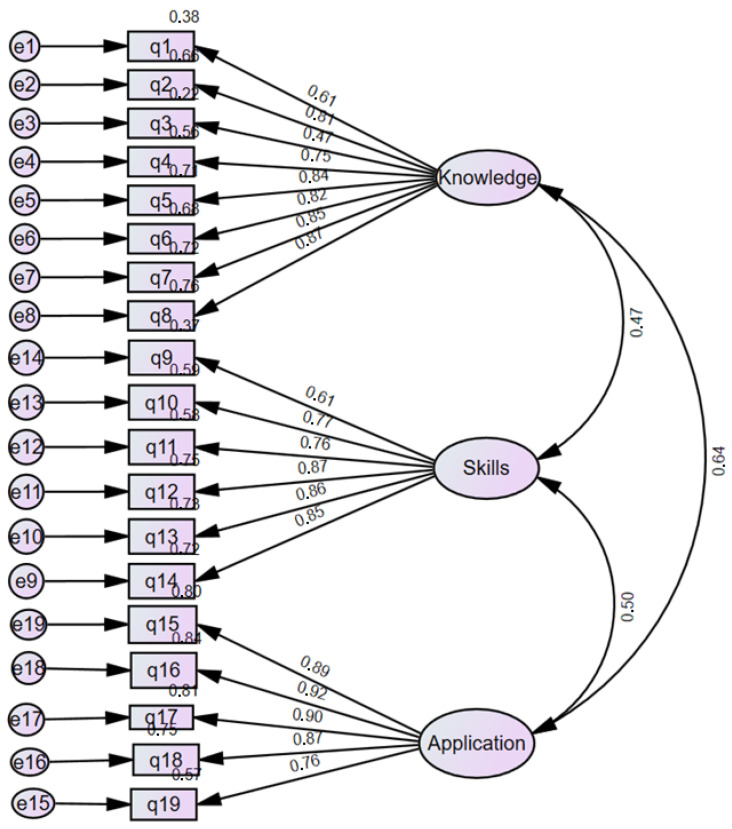
CFA for the RCAIN.

The construct validity of RCAIN was not supported by multifarious key model fit indices, which dropped under the recommended thresholds—thus reflecting the poor total fit of the hypothesized model to the observed data. The Chi-square value for the default model was significant, χ^2^ (df:149) = 371.728, *p* < 0.001, with a CMIN/DF ratio of 2.495, indicating a reasonable fit relative to the degrees of freedom. However, the Goodness of Fit Index (GFI) was 0.718, which is well below the acceptable threshold of 0.90, suggesting poor fit between the hypothesized model and the observed data. The Comparative Fit Index (CFI = 0.865) and Normed Fit Index (NFI = 0.795) are both below the commonly accepted cutoff of 0.90, indicating a suboptimal or weak fit. Finally, the Root Mean Square Error of Approximation (RMSEA = 0.119) with a 90% CI of [0.100, 0.135] is above the threshold of 0.10, which indicates poor model fit. The model showed an acceptable χ^2^/df ratio, but other fit indices (GFI, NFI, CFI, and RMSEA) fell below accepted thresholds, indicating that the overall model fit is marginal to poor (Figure 1).

Furthermore, a moderate, statistically significant positive correlation was found between knowledge and skills (ρ = 0.341, *p* < 0.001), indicating that greater research knowledge was associated with higher research skills. A stronger positive correlation existed between knowledge and application (ρ = 0.542, *p* < 0.001), suggesting that nurses with more research knowledge applied it more often. Skills were also positively correlated with application (ρ = 0.516, *p* < 0.001), meaning that higher skill levels were linked to greater application of those skills.

### 3.3. Comparison of RCAIN Scores by Demographic and Professional Characteristics

As shown in Table 2, knowledge levels differed significantly based on academic qualifications (*p* < 0.001). Nurses without postgraduate or doctoral degrees had significantly lower knowledge scores than master’s students (*p* < 0.001), master’s degree holders (*p* = 0.017), and PhD holders/candidates (*p* = 0.002). Knowledge levels also varied based on participation in educational activities (*p* < 0.001). Those who did not attend workshops or seminars scored significantly lower than those who attended occasionally (*p* = 0.001) or regularly (*p* = 0.004). Research skills varied significantly based on educational level (*p* < 0.001), with PhD holders/candidates scoring significantly higher (*p* = 0.001). Attendance at clinical workshops also influenced skills (*p* = 0.026). Nurses who did not attend had significantly lower skills compared to occasional attendees (*p* = 0.021). Application scores also varied significantly with education (*p* < 0.001). Nurses without postgraduate studies scored lower than PhD candidates/holders (*p* < 0.001) and postgraduate students (*p* = 0.004). Master’s degree holders scored lower than PhD candidates/holders (*p* = 0.048).

A small but significant negative correlation was found between the application of knowledge/skills and years in the current job (ρ = −0.199, *p* = 0.047). Finally, the ability to apply knowledge also varied based on workshop attendance (*p* = 0.042), with non-attendees scoring lower than regular attendees (*p* = 0.040).

## 4. Discussion

The purpose of this study was to assess the research competencies of registered nurses by identifying knowledge and skills relevant to research, as well as the application of their knowledge and skills to their practice. The findings demonstrated that registered nurses showed moderate levels of knowledge, skills, and application of research in clinical practice, as seen by their mean scores in the RCAIN (knowledge: 26.92/40, skills: 22.17/30, and application: 14.89/25). However, it must be stated that, until now, there have not been established RCAIN cutoff points; thus, the characterization of research competencies as “moderate” was based on the relevant position of mean scores within the potential range of each subscale, with values approximating the mid-range of the total attainable score. These moderate scores in the application domain possibly show that, even if nurses have a basic level of research knowledge and skills, the systematic incorporation of research evidence into daily clinical decision making can be inconsistent. In pediatric settings, where care includes complex clinical evaluations and vulnerable populaces, this partial application of evidence could limit the overall potential of evidence-based practice to improve patient outcomes. Our findings highlight the essence of direct strategies that facilitate the translation of research knowledge into routine pediatric nursing practice.

On the other hand, although RCAIN showed high internal consistency across all subscales, the CFA revealed poor overall model fit, suggesting limitations in construct validity within our population-based sample. Various issues could account for this finding, such as the relatively small sample size, the single-center study design, and possible cultural or contextual differences affecting item interpretation; additionally, some RCAIN items may not totally capture research capacity’s dimensions as experienced by Greek pediatric nurses, highlighting a potential item–context mismatch. Accordingly, RCAIN scores from our study must be interpreted with caution, since the instrument may better illustrate general trends in research competencies rather than an entirely validated factorial structure for pediatric nurses in Greece. Furthermore, the results revealed significant, positive correlations among the three domains of the questionnaire, suggesting that higher research knowledge was possibly linked to higher skill levels and more frequent research application in nursing practice. Moreover, education emerged as a basic determinant of research competence; compared to those with no advanced studies, postgraduate and doctoral nurses achieved notably higher scores across all subscales; similarly, compared to those without regular/frequent participation in scientific workshops, lectures or seminars, those disclosing attendance in such activities showed significantly higher levels of knowledge, skills, and application of research. Moreover, a small yet significant negative correlation was seen between years in current position and research knowledge application, suggesting that prolonged tenure with no continued learning could limit the practical application of research findings. Overall, the findings of this study highlight that educational attainment and engagement in research activities are key factors in boosting the research capacity of nurses, as well as promoting nurses’ ability to apply research in daily routine clinical practice.

Our study revealed that registered nurses demonstrated moderate levels of research knowledge, skills, and application of research practices, with education and participation in scientific activities emerging as key factors influencing research capacity. Kengia et al. [19] explored the research capacity and engagement of 462 healthcare workers across 45 public health facilities in Tanzania, and they found low confidence and experience in both qualitative and quantitative research methods, with less than half of participants actively engaged in research. Additionally, some major barriers included limited funding, time, skills, and infrastructure, whereas motivators centered on professional growth, solving health problems, and collaboration, with 92% expressing interest in boosting personal research capacity [19]. Another large study by Xu et al. [20] on 1226 clinical nurses from 14 Shanghai hospitals found that research capacity was at a low-to-medium level, and it was significantly influenced by age, education, professional title, years of experience, department, and position; the most critical factor was the time available for research. The authors concluded that individualized research training, education, and increased engagement in research projects are pivotal to improving nursing research capacity [20]. Another large cross-sectional survey of 3014 nurses from a grade A Chinese tertiary hospital explored self-evaluated research capability and engagement in scientific activities, revealing relatively low research engagement levels, with fewer than 6% of participants having participated in or led nursing research projects, and only 2% having published in international journals [21]. However, in this study, more than 70% of the participants were willing to participate in research, a fact that indicates strong interest but limited practical experience in scientific inquiry [21]. Even if data on pediatric nurses’ research competences are scarce, a Chinese cross-sectional survey similar to our study, which was conducted at a tertiary children’s hospital, estimated the research abilities of 436 pediatric nurses via a self-assessment scale. It reported low research ability levels, while educational level, professional title, and frequency of research training were the strongest independent predictors of greater research competence [22]. A relevant review of the topic also concluded that nurses’ research knowledge and application in pediatric settings exhibit significant variations, with several barriers and facilitators that affect evidence-based practice [23]. The implementation of cultural or practice-level alterations to improve the usage of research can positively influence healthcare outcomes and the quality of pediatric nursing care. Thus, systematic and structured strategies to boost research utilization are required, alongside more studies to estimate both their efficacy and long-term benefits [23]. Moreover, in their scoping review of European nursing research capacity, Egerod et al. [24] noted substantial variability in advanced critical care nursing roles in terms of policies, education, scope of practice, titles and competencies, highlighting the need for precise role definitions, standardized skills, and assessment of patient outcomes. Another Spanish cross-cultural adaptation and validation of a nursing research attitudes instrument revealed high internal consistency but noted a different factor structure through CFA, highlighting the need for future research to prove its applicability in divergent contexts [25]. Similarly, in our research, the RCAIN showed high internal consistency among Greek pediatric nurses, but the CFA indices revealed only marginal to poor construct validity, proposing that cultural adaptation and/or context-specific modifications may be essential to fully depict research competencies in this population. Finally, another systematic review of Italian nurses highlighted a range of obstacles to research engagement, including limited English proficiency, time constraints, poor staff and lack of institutional support, alongside facilitators such as journal reading, expert team support, and university–hospital and international partnerships, offering an initial framework to guide policies that might boost nursing research capacity [26]. However, it must be noted that various studies have used a range of instruments to assess nursing research capacity levels; it is difficult to make comparisons and draw general conclusions due to this divergence in instrumental denominators.

In summary, our study reveals that nursing research capacity is still underdeveloped in Greece; therefore, a well-established research culture and capacity have to be achieved for the nursing profession. Nevertheless, on a global scale, it is important to strengthen clinical nurses’ research capacity via organizational support, mentorship and dedicated research time to foster evidence-based practice, while systematic assessment and directed interventions (such as competency-based training and structured mentorship) are critical to developing research-active nurses and improving the quality of patient care [27]. Furthermore, structured, mentor-led programs can effectively boost clinical nurses’ research competencies, such as their knowledge, skills, and application of evidence-based practice, whereas the incorporation of interactive, cooperative, and resource-driven learning experiences seem critical for the maintenance of improvements in research competence and the promotion of a culture of research engagement in clinical settings [28]. In this context, it is essential to assess pediatric clinical nurses’ research competencies—such as their knowledge, skills, and capability to use research—in order to reveal gaps, tailor training programs, and create and efficient research-capable nurses.

### 4.1. Strengths and Limitations

In terms of its limitations, our study used a relatively small population-based sample, thus limiting the generalizability of our findings to a wider population and representing a single-site sampling frame. In addition, voluntary participation may have introduced response bias, because nurses with greater interest in research, evidence-based practice, or professional development may be more inclined to participate in related studies. Therefore, the recorded levels of research knowledge, skills, and application could be overestimated, and our findings should be interpreted with caution in case of extrapolation to the wider population of pediatric nurses. Moreover, selection biases may have been introduced by the use of convenience sampling, since participation was based on nurses’ willingness and availability. Other desirability biases include the nurses’ honesty and ability to disclose the truth in self-reported questions. Moreover, the cross-sectional design may prevent the establishment of causal relationships between some variables, including education and research competences. Furthermore, the RCAIN model fit indices showed only marginal construct validity in our population-based sample, which suggests a potential partial capture of Greek research capacity dimensions. Additionally, the study was not solely based on quantitative data that might provide deeper insights into facilitators or/and barriers of nursing research capacity. Finally, we did not examine possible confounding factors such as workload, organizational culture, and/or research support, which could have an impact on research competence and engagement.

### 4.2. Future Directions and Implications

Future research should include the assessment of a larger population-based sample and the expansion of the sample by including nurses from different hospitals to allow for multi-site comparisons across diverse pediatric settings. It is also important to perform longitudinal studies to assess alterations in research competencies over time and determine the impact of ongoing educational initiatives. Moreover, the RCAIN questionnaire could be refined and culturally adapted in order to have improved construct validity, as well making it suitable for more populations. Additionally, some organizational factors that may have an impact nurses’ engagement in research activities could be explored, and patient and clinical outcomes linked to higher nursing research competencies could be examined so as to illustrate the practical influence of building research capacity. Finally, future research should focus on re-validating the RCAIN questionnaire by using larger, multi-site population-based samples and conducting exploratory and CFAs to refine the instrument’s structure and strengthen its cultural relevance.

The findings of our research have critical educational implications for pediatric nursing practice. To boost research competencies and evidence-based practice, healthcare organizations and nursing schools could incorporate structured research or relevant modules into continuing education programs. Establishing mentorship programs that pair experienced researchers with clinical nurses, as well as unit-based journal clubs, could also improve the translation of research into daily practice and foster an inquiry culture. Such strategies could strengthen nurses’ confidence and skills in applying research evidence, ultimately enhancing patient care and outcomes in pediatric settings.

## 5. Conclusions

This study assessed the research competencies of nurses in the largest Greek pediatric hospital, as well as the psychometric properties of the RCAIN questionnaire within that context. The findings showed that Greek nurses had moderate levels of research-related knowledge, skills and application, indicating that the existing areas and strengths require further growth. Although the questionnaire showed high internal reliability, the construct validity was limited in our population, suggesting that more refinement and cultural adaptation are essential before its widespread use in pediatric nursing settings in Greece. Attainment in education and engagement in continuous learning (e.g., workshop attendance and post-graduate studies) appeared to be key causal factors of higher research competence. These results highlight the importance of fostering a supportive nursing research culture via targeted education and institutional practices that boost participation in research. Specifically, nursing administrators and educators must consider implementing structured research workshops, offering protected time and institutional support for research engagement, and providing incentives or support for attending and presenting at research conferences to enhance research capacity. Fostering nursing research capacity is critical to advancing evidence-based practice and improving the quality of healthcare delivery.

## Figures and Tables

**Table 1:** Demographic information of the population-based sample.

Demographic Characteristics		
**Gender,** *n (%)*	Female	92 (86.8%)
**Age (years),** Mean (±SD)		44.20 (±10.13)
**Education,** *n (%)*	Bachelor’s Degree in Nursing	12 (11.4%)
	Diploma in Nursing	74 (73.3%)
**Additional Studies,** *n (%)*	Master’s student in Nursing	10 (9.5%)
	Master’s student in a field other than Nursing	4 (3.8%)
	Master’s in Nursing	26 (24.8%)
	Master’s in a field other than Nursing	4 (3.8%)
	Nursing Specialization Certificate	32 (30.2%)
	Doctoral student	2 (1.9%)
	Doctoral degree in Nursing	3 (2.8%)
**Years of work as a nurse,** *Mean (95% CI)*		19.82 (17.56–22.07)
**Years of work in the current organization,** *Mean (95% CI)*		18.66 (16.44–20.89)
**Years of work in the current position,** *Mean (95% CI)*		9.34 (7.54–11.15)
**Work Sector,** *n (%)*	NICU/PICU/ICU	16 (17.6%)
	Surgical Sector	24 (26.4%)
	Pathological Sector	31 (34.1%)
	Laboratory Sector	12 (13.2%)
	Administration	8 (8.8%)
**Position,** *n (%)*	Staff Nurse	78 (73.6%)
	Head Nurse or Nurse Manager	9 (8.5%)
	Staff Nurse with clinical specialization	10 (9.4%)
	Other	9 (8.5%)
**Attendance at clinical workshops, lectures, seminars,** *n (%)*	Yes, regularly	17 (16.2%)
	Yes, sporadically	75 (71.4%)
	No, I almost never attend	10 (9.5%)
	No, I never attend	2 (1.9%)
	Other	1 (1.0%)

**Table 2:** Comparison of RCAIN scores by demographic and professional characteristics of the population-based sample.

Demographic Characteristics	RCAIN Subscales					
	Knowledge		Skills		Application of Knowledge and Skills	
	Mean (95% CI) or Spearman Rho	*p*-Value	Mean (95% CI) or Spearman Rho	*p*-Value	Mean (95% CI) or Spearman Rho	*p*-Value
**Education**		*p* = 0.118		*p* = 0.206		*p* = 0.692
Nursing Diploma	23.20 (17.27–29.13)		21.25 (16.65–25.85)		13.67 (10.28–17.05)	
University Bachelor’s Degree	28.83 (25.17–32.50)		18.29 (13.04–23.54)		14.71 (10.75–18.68)	
**Continuing Education**		***p* < 0.001**		***p* < 0.001**		***p* < 0.001**
No	23.98 (22.08–25.88)		20.74 (19.17–22.31)		12.96 (11.71–14.22)	
MSc(c)	31.92 (30.26–33.58)		24.69 (23.12–26.26)		18.31 (1655–20.06)	
MSc	28.72 (26.61–30.84)		22.34 (19.41–25.28)		15.52 (12.23–17.80)	
PhD(c)/PhD	35.00 (29.77–40.23)		28.50 (26.04–30.96)		22.17 (19.24–25.09)	
**Years of employment in the current position**	rho = −0.182	*p* = 0.070	rho = −0.033	*p* = 0.744	rho = −0.199	***p* = 0.047**
**Clinical Unit**		*p* = 0.109		*p* = 0.602		*p* = 0.913
NICU/PICU/HDI	27.50 (22.32–27.78)		23.06 (19.96–26.16)		16.06 (13.08–19.05)	
Surgical sector	26.88 (25.08–28.67)		20.21 (17.32–23.09)		15.13 (12.81–17.44)	
Medical sector	27.03 (24.70–29.37)		22.94 (21.06–24.81)		14.90 (13.39–16.41)	
Laboratory sector	25.17 (19.83–30.50)		23.25 (20.55–25.95)		13.75 (9.13–18.37)	
Administration	32.88 (29.40–36.35)		18.13 (7.34–28.91)		14.25 (6.41–22.09)	
**Position**		*p* = 0.063		*p* = 0.073		*p* = 0.149
Staff Nurse	25.95 (24.40–27.50)		22.14 (20.79–23.49)		14.32 (13.14–15.50)	
Head Nurse or Nurse Manager	28.11 (21.99–34.23)		24.11 (18.25–29.97)		17.22 (13.75–20.70)	
Nurse with clinical specialization	28.40 (22.77–34.03)		24.20 (20.83–27.57)		17.00 (13.04–20.96)	
Other	32.56 (28.87–36.24)		18.22 (11.62–24.82)		15.11 (9.40–20.82)	
**Attendance at clinical workshops, lectures, seminars**		***p* < 0.001**		***p* = 0.026**		***p* = 0.042**
Yes, regularly	30.65 (27.80–33.50)		21.29 (17.48–25.11)		16.18 (12.90–19.45)	
Yes, sporadically	27.29 (25.74–28.84)		22.67 (21.18–24.15)		15.05 (13.84–16.27)	
No, I almost never attend/I never attend	19.92 (15.63–24.20)		20.00 (17.83–22.17)		11.67 (8.69–14.64)	

## Data Availability

Data sharing is not applicable to this article. The data are not publicly available due to restrictions. They contain information that could compromise the privacy of the participants.

## Public Involvement Statement

No public involvement in any aspect of this research.

## Guidelines and Standards Statement

This manuscript was drafted in accordance with the Strengthening the Reporting of Observational Studies in Epidemiology (STROBE) Statement (cross-sectional checklist) for observational cross-sectional research.
